# Supplementary material for: Near-term forecasting of Covid-19 cases and hospitalisations in Aotearoa New Zealand
Source: PLoS Comput Biol. 2024 Jan 8;20(1):e1011752. doi: 10.1371/journal.pcbi.1011752 (PMC10798620; doi:10.1371/journal.pcbi.1011752)
Supplement: Attachment — Submitted filename: response to reviewers.docx [file pcbi.1011752.s002.docx]

**Author responses to reviewer comments**

**Reviewer #1:** The paper describes and evaluates a methodology for forecasting COVID-19 cases and hospital occupancy in Aotearoa New Zealand. The description of the model is comprehensive and concise. The treatment of input data, including the exclusion of censored data points is well described. The use of CRPS and bias scores to measure forecasting performance is appropriate and subsequent results well presented. I note a few points of suggestion that may improve the work as it stands:

**Reviewer comment:** 1. The magnitude and pattern of data censoring in Supplementary Figure 1b is hard to discern due to the small size of the plot. Increasing the size of this plot (or otherwise changing the data presentation) would help clarify this.

**Response:** We have made the plots larger in Supplementary Figure 1b to make this clearer.

**Reviewer comment:** 2. Line 146 – “of cased” to “of cases”

**Response:** Fixed.

**Reviewer comment:** 3. Line 190 – “as independent Gaussian process” to “as independent Gaussian processes” (or “as an independent Gaussian process”?)

**Response:** Fixed.

**Reviewer comment:** 4. Discussion of the calibration of forecast outputs (e.g. line 263, 275) would be strengthened by the inclusion of a result quantitatively evaluating this (e.g. reporting the percentage of observations within the 90% intervals or a probability integral transform plot [1]).

[1] Diebold, F. X., Gunther, T. A., & Tay, A. S. (1998). Evaluating density forecasts with applications to financial risk management. International Economic Review, 39(4), 863.

**Response:** We have now reported the percentage of observations that fell within the 90% prediction intervals for cases, admissions and occupancy in the 21-day ahead forecast (see line 281).

**Reviewer #2:** This paper was easy to read and straightforward. It’s nice to see this type of model in use in a public health setting. The comments I’m providing are mostly suggestions and not “make or break.” Thanks!

Major comments
**Reviewer comment:** Did you all consider modeling the hospital admissions and occupancy directly, instead of modeling them as a portion of cases? The only reason I bring this up is because that type of data is much more reliable than case data and I’d imagine it would be more straightforward to model them directly.

**Response:** This is a good point and we have added some commentary in the Discussion on the relative advantages and disadvantages of our approach compared to modelling hospitalisation directly (see lines 373-380).

**Reviewer comment:** Did you have any issues with particle convergence (i.e., where the effective number of particles declines to some nominal number due to large differences in likelihood-based weights)? How did you deal with those? If not, how did you avoid particle convergence?

**Response:** We did not encounter issues with particle degeneracy. We have now added a supplementary figure (S8) showing the number of unique particles and referred to this in the Results section (line 313-318).

**Reviewer comment:** I was wondering a bit on the rationale for some of the parameter choices in Table 2. Specifically with the dispersion parameter values being quite high. Did you consider just using a Poisson model instead of a Negative Binomial model for cases w/ a dispersion parameter of 100? If you have any testing that you performed in picking the parameter values, it would be interesting (although not necessary) to see examples of how the model results differ with lower dispersion parameters.

**Response:** We have now added a note that for the chosen values of the dispersion coefficients, the distributions for daily cases and admissions are close to Poisson, but we retain the negative binomial model for generality and flexibility to other datasets (see line 246-250). We have also added a new results for a sensitivity analysis of the dispersion parameters and the random walk standard deviation parameter (see lines 302-312 and Supplementary Figures S5-S7).

Minor comments
**Reviewer comment:** For figure 3, could you please add in the legend what the red color denotes?

**Response:** Done.

**Reviewer comment:** For figure 2 and 3 (and any w/ that same set up), I think it would be more useful to see the uncertainty visualized as a shading gradient rather than lines for the quantiles. Additionally, I don’t think it’s necessary to have so many quantiles shown, perhaps showing just 5, 25, median (as a thicker line), 75, 95 would be good enough? The way that those figures are set up right now are busy and somewhat difficult to analyze.

**Response:** We have tried this and found that it made the Figures harder to read. Instead, we have ensured that the grey curves are ‘underneath’ the data, and showing the data as points rather than open circles. This enables the location of the data to be more precisely seen with respect to the forecast quantiles, and prevents the markers from overlapping and cluttering up the plot. We think this has improved the legibility and clarity of the plots.

**Reviewer comment:** In the discussion, it would be useful to comment more on instances where you think the model failed or did poorly on the forecast scoring. For example, why do you think the hospital admissions and hospital occupancy models are positively and negatively biased? What does that mean practically speaking and how does it affect the interpretation of the results?

**Response:** We have added a comment on why we think the admission forecast is positively biased whereas the case forecast is not (see line 299-301). We have also added some general discussion of the scoring results and the implications for practical applications of the model (line 353-357).

**Reviewer #3:** The paper presented a forecasting model suited to New Zealand's unique COVID-19 surveillance systems. This combined a semi-mechanistic stochastic model of cases and a Gaussian process regression model of the age-specific case hospitalisation ratio. Hidden states related to cases were fit using a particle filter, age-specific lengths of stay in hospital were directly estimated empirically, relative number of cases by age group and case hospitalisation ratio through time were fit to Guassian process regression models.

The approach to modelling is sound for the forecast horizon of 3 weeks (noting that susceptible depletion will play a role over longer time horizons) and the model provides reasonable forecasts of New Zealand daily cases and hospital admissions. The approach here is nice in that it present a flexible model that avoids the added complexity of features such as vaccination or past waves of infection. I have some minor suggestions for improvements, but otherwise this paper is appropriate for publication.

**Reviewer comment:** (1) Figure 2 is confusing, the authors describe the particles as relating to states I, R and Z and these states being fit to case data, so quantiles for the case trajectory do not make sense in regions where there is data. My guess is the authors have sampled from the relevant negative binomial distribution given Z_t's of each particle, but this is not described.

**Response:** Yes that is the correct interpretation – we have now clarified this in the caption for Figure 2.

**Reviewer comment:** (2) In Figure 1(b) the estimated 40-50 age group case hospitalisation ratio does not track the data particularly well in comparison to the other age groups. Some comments on this in the text would be helpful.

**Response:** We have added a comment to the text that the fitted Gaussian process regression for the CHR in the 40-50s group represents the data as a lower frequency signal with higher noise compared to the other groups (lines 263-265).

**Reviewer comment:** (3) Pg 6. has typographical error 'cased'

**Response:** Fixed.

**Reviewer comment:** (4) Pg 7. Z_t is described as the 'expected number of cases reported on day t' but this isn't quite accurate as Z_tw_{i[t]} is the expected number of cases on day t

**Response:** We have now clarified this (see line 167).

**Reviewer comment:** (5) Pg8. N is not defined

**Response:** Fixed (see line 172).

**Reviewer comment:** (6) Pg9. Equation 11 should not have the A_{t'}

**Response:** Fixed.
